# Fine-grained topographic organization within somatosensory cortex during resting-state and emotional face-matching task and its association with ASD traits

**DOI:** 10.1038/s41398-023-02559-3

**Published:** 2023-07-27

**Authors:** Christina Isakoglou, Koen V. Haak, Thomas Wolfers, Dorothea L. Floris, Alberto Llera, Marianne Oldehinkel, Natalie J. Forde, Bethany F. M. Oakley, Julian Tillmann, Rosemary J. Holt, Carolin Moessnang, Eva Loth, Thomas Bourgeron, Simon Baron-Cohen, Tony Charman, Tobias Banaschewski, Declan G. M. Murphy, Jan K. Buitelaar, Andre F. Marquand, Christian F. Beckmann

**Affiliations:** 1 Donders Institute for Brain, Cognition, and Behavior, Radboud University, Nijmegen, Netherlands; 2 Department for Cognitive Neuroscience, Radboud University Medical Center, Nijmegen, Netherlands; 3 Department of Psychology, University of Oslo, Oslo, Norway; 4 Methods of Plasticity Research, Department of Psychology, University of Zurich, Zurich, Switzerland; 5 Department of Forensic and Neurodevelopmental Sciences, Institute of Psychiatry, Psychology and Neuroscience, King’s College London, London, United Kingdom; 6 Roche Pharma Research and Early Development, Neuroscience and Rare Diseases, Roche Innovation Center Basel, F. Hoffmann–La Roche Ltd., Basel, Switzerland; 7 Autism Research Centre, Department of Psychiatry, University of Cambridge, Cambridge, United Kingdom; 8 Department of Psychiatry and Psychotherapy, Central Institute of Mental Health, Medical Faculty Mannheim, University of Heidelberg, Mannheim, Germany; 9 Department of Applied Psychology, SRH University, Heidelberg, Germany; 10 Human Genetics and Cognitive Functions, Institut Pasteur, Université de Paris, Paris, France; 11 Department of Psychology, Institute of Psychiatry, Psychology and Neuroscience, King’s College London, London, United Kingdom; 12 Department of Child and Adolescent Psychiatry, Central Institute of Mental Health, Medical Faculty Mannheim, University of Heidelberg, Mannheim, Germany; 13 Karakter Child and Adolescent Psychiatry University Centre, Nijmegen, Netherlands; 14 Department of Neuroimaging, Institute of Psychiatry, Psychology & Neuroscience, King’s College London, London, United Kingdom; 15 Centre for Functional MRI of the Brain, University of Oxford, Oxford, United Kingdom

**Keywords:** Diagnostic markers, Neuroscience, Autism spectrum disorders

## Abstract

Sensory atypicalities are particularly common in autism spectrum disorders (ASD). Nevertheless, our knowledge about the divergent functioning of the underlying somatosensory region and its association with ASD phenotype features is limited. We applied a data-driven approach to map the fine-grained variations in functional connectivity of the primary somatosensory cortex (S1) to the rest of the brain in 240 autistic and 164 neurotypical individuals from the EU-AIMS LEAP dataset, aged between 7 and 30. We estimated the S1 connection topography (‘connectopy’) at rest and during the emotional face-matching (Hariri) task, an established measure of emotion reactivity, and accessed its association with a set of clinical and behavioral variables. We first demonstrated that the S1 connectopy is organized along a dorsoventral axis, mapping onto the S1 somatotopic organization. We then found that its spatial characteristics were linked to the individuals’ adaptive functioning skills, as measured by the Vineland Adaptive Behavior Scales, across the whole sample. Higher functional differentiation characterized the S1 connectopies of individuals with higher daily life adaptive skills. Notably, we detected significant differences between rest and the Hariri task in the S1 connectopies, as well as their projection maps onto the rest of the brain suggesting a task-modulating effect on S1 due to emotion processing. All in all, variation of adaptive skills appears to be reflected in the brain’s mesoscale neural circuitry, as shown by the S1 connectivity profile, which is also differentially modulated during rest and emotional processing.

## Introduction

Sensory processing (SP) atypicalities constitute a prominent feature in the clinical presentation of autism spectrum disorders (ASD), affecting more than 90 percent of the diagnosed individuals [1]. ASD is primarily characterized by social, communication, and/or restricted/repetitive behaviors [2], with SP differentiation only assessed as part of the latter according to the latest version of the Diagnostic and Statistical Manual of Mental Disorders (DSM-5).

Sensory processing difficulties are common across the ASD continuum imposing a strong need for understanding how these contribute to or interact with other core ASD symptoms. Some studies have previously shown that SP atypicalities are related to overall ASD severity - although more so at younger ages - as well as social difficulties [3], level of daily functioning [4, 5], and adaptive behavior [6]. Essentially, difficulties with sensory processing may amplify social interaction difficulties [4], or be at their root. For instance, SP disruption is predictive of maladaptive behaviors both in cross-sectional [6, 7], as well as in longitudinal studies [8]. Moreover, tactile processing dysfunction has been explicitly linked to social difficulties in individuals with ASD [9], highlighting the importance of touch in early development for forming relationships [10]. Despite the evidence suggesting a relationship between sensory processes and higher-order functions, such as those mentioned above, the neural underpinnings that could potentially link the two remain unclear.

In this work, we focus on the primary somatosensory cortex (S1), which is a core brain area for processing somatosensory information [10, 11]. While mostly known for the processing of tactile information and pain [12, 13], growing evidence supports the notion that multisensory integration, which is essential for the perception of complex social information and appears to diverge in autistic individuals relative to neurotypicals [1], takes place within S1 and other sensory cortices as well [14, 15]. Furthermore, autistic adults appear to have a disrupted cortical representation of their face and hand, but there is a further need to understand the involvement of this region in ASD, and has been the main objective of this work.

Moreover, the data at hand gave us the opportunity to study S1 not only at rest, but also under different tasks probing various aspects of cognition, with emotional processing being one of them. S1 has been linked before to all stages of emotional processing (i.e. Identification of emotional significance in a stimulus, generation of emotional states, and regulation of emotion) due to its direct and indirect connections through the insula with the amygdala [10]. Previous studies on participants with mood disorders or lesions in the somatosensory cortex, as well as in neurotypical individuals, report structural and functional changes in their somatosensory cortex in response to emotional stimuli, but this has not been done as of yet and to the best of our knowledge in the context of ASD [10]. It has been nevertheless suggested that lower-level tactile perception could be intact in autistic individuals, but differences in sensory under- or over-responsivity might be attributed to emotional difficulties instead [11]. The question of whether and how S1 is being modulated in the presence of emotional load constitutes a secondary, but equally important, target of this work. Our understanding of lower-order atypicalities of the somatosensory cortex, present but not entirely unique in ASD, and their co-occurence with other distressing higher-order symptoms, such as emotion dysregulation, may benefit from this aim.

Neurotypical or neurodivergent brain functioning of S1 can be understood and characterized through its topographic organization [16]. This principle governs the S1 organization; adjacent parts of the body are represented in adjacent positions in the cortex. The somatotopic organization is typically being assessed through task-evoked activity where distinct areas of the body are being stimulated, resulting in distinct activation foci in S1 in alignment with what is known as sensory homunculus. A few methods have been proposed to directly capture the gradual nature of brain topographies; one of them is the ‘connectopic mapping’ approach [17]. This method extends the predominant in literature approach of estimating connectivity between regions that have been defined based on hard parcellation schemes under the assumption of piecewise constant connectivity patches and manages to capture smoothly-varying ‘connection topographies’ (‘connectopies’) [18], without violating the region’s, in this case S1’s, exhibited functional multiplicity [19]. In previous work, it was established that the topographic map of the primary motor cortex – a brain region with a well-known topographic organization as well - closely maps to its somatotopic organization [17]. In the present work, S1 provides the ground for studying the neural underpinnings of low-level sensory processing, as well as their potential link to higher-order ASD features. Notably, such an examination ought to take place at an individual level, so that the mapping constructed between behavioral and biological scores takes into account the heterogeneity of the ASD condition, reported and discussed elsewhere [20], instead of relying on a case-control approach that could conceal such potential relationships [21], with the behavioral and/or clinical score variation present in neurotypical individuals also being taken into consideration.

Here, we investigate (1) whether S1’s topographic organization maps onto clinical scores measuring sensory processing, along with other daily life skills and abilities, reported to be affected in ASD, and (2) how S1’s “rest topography” varies with emotional processing demands, in other words, whether it is modulated by an emotion-eliciting task. To address these aims we applied connectopic mapping to the S1 cortex of neurotypical and autistic individuals to (i) find the association between the spatial characteristics of the estimated connection topographies and dimensional symptom scores across all individuals, and (ii) assess whether those spatial characteristics are different in an emotional processing task, relative to the resting-state.

## Subjects and methods

### Neuroimaging data

The dataset that was used for the current analysis was part of the EU-AIMS LEAP project, a multi-site European collaborative effort with its main aim to identify biomarkers associated with ASD. The study design is described extensively in an earlier publication by Loth et al. [22] along with a comprehensive overview of the clinical characterization of the cohort [23] (for data acquisition details, see Supplementary Material).

Resting-state functional MRI (rfMRI) scans were available for 656 participants, to which we applied a set of stringent quality control exclusion criteria. Participants with a structural brain abnormality (not clinically relevant; *n* = 17), an incomplete rfMRI scan (*n* = 8), excessive head motion during the rfMRI scan (mean frame-wise displacement > .7 mm[*n* = 29; 5% highest average head motion in our sample], max frame-wise displacement> 3.8 mm[n = 38; 1 voxel size]), or insufficient brain coverage (*n* = 24) were excluded (*n* = 145). We further excluded participants with insufficient variance of voxels in the selected region of interest and/or poorly estimated connectopies (*n* = 107; see Section ‘Connectopic Mapping’). This resulted in the inclusion of 404 participants with rfMRI scans.

For the emotional processing task, we used the Hariri emotion processing fMRI task [24] performed by 287 participants. This task followed a block design paradigm and each block included 6 trials of the same task (face or shape). Each of the two runs included 3 face blocks and 3 shape blocks, with 8 s of fixation at the end of each run. The participants were presented with blocks of trials that either ask them to decide which of two faces presented on the bottom of the screen match the face at the top of the screen(experimental condition), or which two shapes presented at the bottom of the screen match the shape at the top of the screen (control condition). The faces have either an angry or fearful expression and each lock is preceded by a 3000 ms task cue, so that each block is 21 s including the cue. After quality control, we included 249 participants in the analysis.

Demographic and clinical characteristics of our sample for rfMRI and the Hariri task can be found in Table 1 and Table 2, respectively.

**Table 1:** Participant demographic and clinical characteristics for neurotypical (NT) and autistic (ASD) participants with rfMRI.

	*n*	All (*n* = 404) (Mean ± SD)	ASD (*n* = 240) (Mean ± SD)	NT (*n* = 164) (Mean ± SD)
*Demographic measures*				
Autistic:Neurotypical	404	240:164		
Biological sex (female:male)		128:276	62:178	66:98
Age in years		16.74 ± 5.3	16.95 ± 5.25	16.43 ± 5.37
Full-scale IQ	401	102.29 ± 18.93	100.08 ± 19.03	105.5 ± 18.37
Handedness (left-handed:right-handed:ambidextrous)^a^	347	43:293:11	29:173:8	14:120:3
*Clinical measures*				
SSP^b^	222	153.55 ± 29.25	139.03 ± 26.73	177.86 ± 12.19
SRS-2^c^	354	60.92 ± 15.14	69.74 ± 11.82	46.76 ± 6.9
RBS-R	228	14.15 ± 13.59	16.35 ± 13.85	3.49 ± 3.8
Vineland-II Communication	258	78.16 ± 18.73	75.03 ± 15.34	89.96 ± 24.91
Vineland-II Daily living	256	76.82 ± 18.66	73.27 ± 15.88	90.11 ± 22.16
Vineland-II Socialization	254	76.53 ± 21.62	71.09 ± 16.48	97.15 ± 26.14
Vineland-II ABC	252	75.27 ± 18.34	71.02 ± 13.53	91.26 ± 24.42
ADI-R (Social)	229	-	16.53 ± 6.77	-
ADI-R (Communication)	228	-	13.37 ± 5.57	-
ADI-R (Restricted and Repetitive behaviors)	217	-	4.5 ± 2.44	-
ADOS-2^d^	236	-	5.15 ± 2.68	-

**Table 2:** Participant demographic and clinical characteristics for neurotypical (NT) and autistic (ASD) participants with Hariri task fMRI.

	*n*	All (*n* = 249) (Mean ± SD)	ASD (*n* = 124) (Mean ± SD)	NT (*n* = 125) (Mean ± SD)
*Demographic measures*				
Autistic:Neurotypical	249	124:125		
Biological sex (female:male)		75:174	39:85	36:89
Age in years	249	17.49 ± 5.48	17.79 ± 5.7	17.19 ± 5.26
Full-scale IQ	248	107.42 ± 13.97	107.06 ± 15.9	107.78 ± 11.78
Handedness (left-handed:right-handed:ambidextrous)^a^	214	23:185:6	11:94:3	12:91:3
*Clinical measures*				
SSP^b^	134	158.71 ± 28.44	143.45 ± 27.87	178.71 ± 12.16
SRS-2^c^	227	57.18 ± 14.8	67.56 ± 13.16	46.52 ± 6.47
RBS-R	119	11.94 ± 12.2	14.54 ± 12.63	3.07 ± 3.35
Vineland-II Communication	135	81.18 ± 17.99	77.37 ± 15.79	105.94 ± 9.97
Vineland-II Daily living	134	80.15 ± 17.47	76.43 ± 15.24	104.11 ± 10.68
Vineland-II Socialization	133	79.07 ± 20.43	73.81 ± 16.07	112.67 ± 11.28
Vineland-II ABC	132	77.98 ± 16.97	73.52 ± 13.27	106.22 ± 8.35
ADI-R (Social)	119	-	16.06 ± 6.61	-
ADI-R (Communication)	118	-	13.03 ± 5.62	-
ADI-R (Restricted and Repetitive behaviors)	112	-	4.28 ± 2.61	-
ADOS-2^d^	122	-	5.2 ± 2.7	-

*SD* standard deviation, *IQ* intelligence quotient, *SSP* short sensory profile, *SRS-2* social responsiveness scale-2, *Vineland-II ABC* Vineland-II adaptive behavior composite, *ADI-R* Autism Diagnostic Interview-Revised (only available for autistic individuals), *ADOS-2* Autism Diagnostic Observation Schedule-Second Edition (only available for autistic individuals).

^a^Handedness information missing for 57 participants (rfMRI) and for 35 participants (Hariri).

^b^Total score (parent-report).

^c^Total T-score (combined parent- and self-report). Parent-report score prioritized over self-report score.

^d^Combine Calibrated Severity Scores (CSS) (Based on full data & imputed data).

### fMRI preprocessing

Preprocessing of both the rfMRI and task fMRI was performed using a standard preprocessing pipeline that included tools from the FMRIB Software Library (FSL version 5.0.6; http://www.fmrib.ox.ac.uk/fsl). For the rfMRI data, we initially recombined the three rfMRI scan echoes using echo-time weighted averaging. Preprocessing included removal of the first five volumes to allow for signal equilibration, primary head motion correction via realignment to the middle volume using MCFLIRT [25], global 4D mean intensity normalization and spatial smoothing with a 6 mm FWHM Gaussian kernel. Then, ICA-AROMA was used to identify and remove secondary motion-related artifacts [26, 27]. Next, nuisance regression was applied to remove signal from white matter and CSF, and a 0.01 Hz high-pass filter was applied to remove very low-frequency drifts in the time-series data. To preserve the broad-band characteristics of functional connectivity data no low-pass filtering was applied [28]. The fMRI images of each participant were co-registered to the participants' high-resolution T1 anatomical images via boundary-based registration (BBR) implemented in FSL FLIRT [25]. The T1 images of each participant were registered to MNI152 standard space with FLIRT 12-dof linear registration [29], and further refined using FNIRT non-linear registration (10 mm warp, 2 mm resampling resolution) [25]. Finally, we brought all participant-level fMRI images to 2 mm MNI152 standard space, in which all further analyses were conducted.

### Clinical measures

The autistic participants have received clinical diagnosis according to the DSM-IV, ICD-10, or DSM-5 criteria. Furthermore, additional dimensional measures of sensory processing, social and adaptive functioning for both neurotypical and autistic participants were available. The *Short sensory profile* (SSP) [30] was used to assess sensory processing atypicalities across 38 items from which an overall raw score was derived that reflects sensory processing across multiple sensory domains. The Repetitive Behaviors Scale-Revised (RBS-R) was used to measure the repetitive behavior observed in individuals. The *Social Responsiveness Scale-Second Edition* (SRS-2) [31] was used to assess distinctions in social behavior associated with ASD across a 65-item rating scale. The overall T-scores from parent and self-reports (for neurotypical adults, SRS-2 self-report version of the questionnaire was administered, while for the rest a parent-reported symptom questionnaire was available) are reported here. Finally, the *Vineland Adaptive Behavior Scales, Second Edition* (Vineland-II) [32] a semi-structured parent interview, was used to assess the adaptive functioning of participants across three domains: communication, socialization, and daily living skills. For a more extensive description of all the existing clinical measures of this project, check the work from Charman et al. [23].. The Autism Diagnostic Interview-Revised (ADI-R) and Autism Diagnostic Observation Scale (ADOS-2) were the two diagnostic instruments used in the diagnosis of autistic individuals. The studies reported in the introduction used the aforementioned dimensional measures SRS-2 and Vineland-II to explore the link between sensory atypicalities and difficulties in social life or adaptive functioning, respectively. Additionally, repetitive behaviors, another key feature of ASD, have also been linked with different aspects of sensory atypicalities (e.g., heightened level of tactile seeking) [33].

The differential availability of the measures led to a limited subgroup of participants examined for each one (Table 1 and Table 2). However, repeating the analysis using imputed measures led to similar conclusions. The imputed measures were estimated using Extra Tree multivariate regression, a method that outperformed all other imputation strategies evaluated in the specific dataset (e.g., K-nearest neighbors, Bayesian Ridge Regression, mean and median variable imputation) [34].

### Connectopic mapping

We selected the post-central gyrus based on the Harvard–Oxford atlas (available as part of FSL [35]) as our region of interest. Based on this ROI we estimated connection topographies (‘connectopies’) [17] separately for each participant, hemisphere, and imaging measure (rfMRI, Hariri task) using *congrads* (publicly available at https://github.com/koenhaak/congrads). This analysis approach involves three main steps and is summarized in Fig. 1 of the Supplementary Material. Briefly though, first we obtain the connectivity fingerprint of each somatosensory voxel computed as the Pearson correlation between the voxel-wise time series from the predefined ROI and the losslessly SVD-transformed time series from all gray-matter voxels outside the ROI. We then insert the fingerprint matrix to a manifold learning algorithm (Laplacian eigenmaps) to obtain a set of connection topographies. The derived connectopies represent how the connectivity between the target ROI and the rest of the cortex varies topographically within the specified ROI. Finally, we fit a spatial statistical model (a ‘trend surface model’) to each connection topography. In this work, we focus on the first and primary connectopy derived from this method, which closely follows the somatotopic organization. The position along the colorbar of the connectopy reflects the similarity between the connectivity profile with similar colors indicating similar functional connectivity profile.

**Fig. 1:**
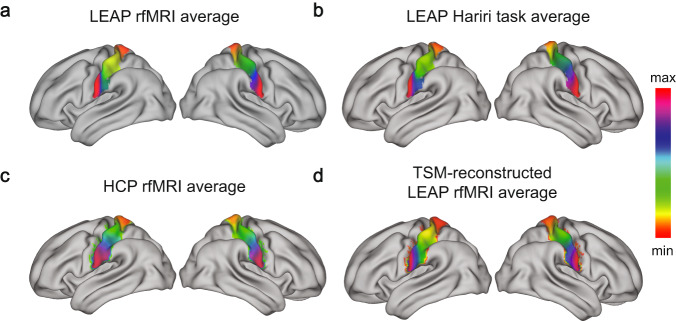
Somatosensory connectopies in LEAP and HCP datasets. Illustration for both hemispheres of the **a** average S1 connectopy at rest in the LEAP dataset, the **b** average S1 connectopy during the Hariri task in the LEAP dataset, the **c** HCP S1 connectopy, used as a reference, and the **d** TSM-reconstructed (6^th^ model order) average S1 connectopy at rest in the LEAP dataset. The colorbar indicates the position along the primary mode of connectivity change - similar colors represent similar connectivity patterns.

Trend Surface Modelling (TSM) was applied to the connectopies to obtain a lower-dimensional representation which facilitates further statistical inference [17]. By applying TSM to our connectopies, we obtain a set of parameters (‘TSM coefficients’) that correspond to polynomial coefficients of varying order along 3D Euclidian axes and summarize the connectopies as a set of spatial polynomial basis functions. The polynomial degree, i.e., the number of TSM coefficients, controls the granularity of the representation of the connectopic map and was chosen based on the value that minimized the model’s Bayesian information criterion (BIC) while, at the same time, maximizing its explained variance (EV).

We further used a reference connectopy derived from the well-known excellent quality rfMRI dataset Human Connectome Project (HCP) in which the somatotopic organization of the primary motor cortex has been mapped before [17]. We compared the connectopies between the two datasets, so that we get an estimation of the quality of the obtained LEAP connectopies, as well as of the validity of the main axis of connectivity change that we observe. To sensitize our analyses to subtle variations within connectopies, while ensuring that poor-quality connectopies do not unnecessarily inflate the polynomial degree, individuals whose connectopies correlated less than *r* = 0.5 with the reference HCP connectopy were excluded from further analysis (*n* = 99 [neurotypical:autistic=54:45]; see ‘Data preparation for connectopic mapping’ in Supplementary Material).

Additionally, we estimated the projection maps of the connectopies onto the rest of the brain by using dual regression to map the whole-brain connectivity changes associated with the subtle variations within the ROI [36]. Dual regression allows us to obtain subject-wise spatial maps based on group-ICA components, avoiding the pitfall of comparing components that are not well-matched between subjects, but rather derive from differences in decomposition across them.

### Association with clinical measures and comparison between connectopies

The Pearson’s correlation coefficient between the TSM coefficients of the connectopies and the available clinical measures was calculated. We additionally controlled for biological sex, age, full-scale IQ and site via OLS multiple regression. Separate GLM analysis on raw connectopies was conducted using as explanatory variables ASD diagnosis, sex, age, and the clinical and behavioral scores reported in Table 1, as a complementary analysis to ensure the polynomial fitting of the connectopies does not limit our results. *P*-values have been corrected for multiple comparisons via the Bonferroni-Holm method (FWER = 0.05). We compared the connectopies between groups of neurotypical and autistic individuals, as well as between rest and task, using *t*-tests, independent and paired samples, respectively. The spatial correlation between connectopies was calculated in a voxel-wise manner. The implementation code is publicly available at https://github.com/cisakoglou/congrads_sensory_leap.

## Results

### Average connectopies estimated across individuals during rest and the Hariri task

For the modeling of the S1 connectopy the 6^th^ order was selected for our model (see Supplementary Fig. 2). This entailed the representation of each connectopy with a set of 18 parameters (x, y, z, x^2^, y^2^, z^2^, x^3^, etc.) during both rest and task. That is, spatial variation within the ROI was modeled through a trend surface that was described by polynomials up to 6^th^ order in x,y, and z direction.

The average connectopy across all individuals – autistic and neurotypical individuals combined – is illustrated in Fig. 1a for rest and Fig. 1b for task. After visual inspection, we observe the similarity with the average connectopy estimated from HCP (Fig. 1c). The voxel-wise spatial correlation between the HCP connectopy and the one during each imaging measure (task and rest) was estimated (HCP-rest: (left hemisphere) Pearson’s *r* = 0.83, (right hemisphere) Pearson’s *r* = 0.94; HCP-task: (left hemisphere) Pearson’s *r* = 0.89, (right hemisphere) Pearson’s *r* = 0.96; *p*-values < .001 for all four comparisons).

The average S1 connectopy along the dorsoventral axis of each hemisphere was reproduced across both conditions and datasets with similar reconstructions of the connectopies for all (see reconstruction for the average S1 connectopy during rest in Fig. 1d) and matches the somatotopic organization.

### Association between S1 connectopies and ASD dimensional measures

During the Hariri task, a significant correlation was found between two TSM coefficients (z^2^, z^4^) reconstructing the S1 connectopy of the left hemisphere and Vineland-II Daily Living scores (see Supplementary Material). Correlations with the Vineland-II Socialization and Adaptive Behavior Composite (ABC) standard scores were also high but did not survive multiple comparisons correction. The complete results of the correlation analysis during the Hariri task can be found in Supplementary Figs. 4 and 5. We performed a sensitivity analysis to ensure those results are not driven by edge effects by repeating the analysis on the eroded connectopies of 50 randomly selected participants and the TSM coefficients were not found to differ significantly between the original and the eroded connectopies (see Supplementary Fig. 14). This resulted in almost identical associations with the behavioral scores as well.

We conducted a separate GLM analysis on raw S1 connectopies to examine the localization of the above correlation and ensure that the observed effects are not driven by limitations from the polynomial modeling. As reported in Supplementary Figs. 8–10, Vineland-II Daily-Living, Socialization and Communication scores survived correction, and the association was mostly located at the S1/M1 boundaries.

During rest, some TSM coefficients (see Supplementary Material) were found to be correlated with Vineland-II Daily Living and Vineland-II ABC scores. However, these correlations did not withstand multiple comparisons correction. We also correlated TSM coefficients with SSP, SRS-2, and Vineland-II Socialization, and no significant relationship was found. The complete results of the correlation analysis during rest can be found in Supplementary Figs. 6 and 7.

**Fig. 2:**
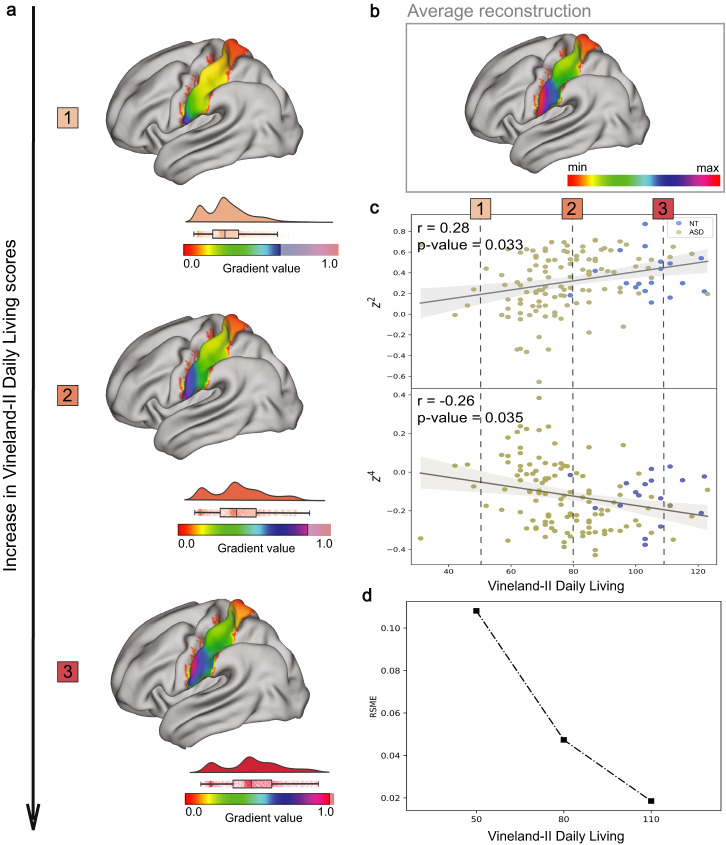
Visualization of the change in the connectivity profile within left hemisphere’s S1 during the Hariri task as a function of the Vineland-II Daily Living scores. In **a** we visualize the reconstructions of the connectopies that correspond to the three evenly spread selected points across the Vineland-II Daily Living scale highlighted in (**c**). The arrow indicates the direction towards which the Vineland-II Daily Living scores increase - reflecting higher functioning. The gradients’ color range varies along this increase in the functioning scale which is illustrated by a raincloud visualization shifting its max value more towards the extreme of the color range in each of the reconstructions. We further illustrate in **b** the TSM-reconstructed average S1 connectopy of the left hemisphere during the Hariri task, in **c** the scatterplots of the two TSM coefficients z^2^ and z^4^ reconstructing the S1 connectopies and which were found to be significantly associated with Vineland-II Daily Living scores, and in **d** the Root Mean Square Error (RMSE) between the reconstructed S1 connectopy of each of the three aforementioned selected points from Vineland-II Daily Living scale and the average S1 reconstructed connectopy. The decrease of RSME observed with the increase of the Vineland-II Daily Living scores demonstrates how similar the S1 connectopy gets with the average one as the individuals’ functioning gets higher.

In addition, we aimed to understand how the S1 connectopy during the Hariri task changes as a function of variation within clinical measures. Having already obtained the TSM coefficients reconstructing the S1 connectopy and the respective clinical measure with which they were associated, we visualized the clinical associations of the underlying topography in the following manner; We derived TSM-reconstructed connectopies at three evenly spread points across the Vineland-II Daily Living scale (Fig. 2a) by fitting their original reconstructions to the average TSM-reconstructed connectopy (Fig. 2b) and obtaining their respective residual connectopies. The points across the Vineland scale that were selected are highlighted as 1,2 and 3 in Fig. 2c*.*

We further calculated the root-mean-square error (RMSE) between the TSM coefficients reconstructing the average connectopy in the z direction – this includes the coefficients that were found to correlate significantly with Vineland-II scores – and the TSM coefficients reconstructing the connectopy and corresponding to each of the selected points on the Vineland-II Daily Living scale highlighted in Fig. 2c*.* In Fig. 2c, we have shown how the RMSE decreases as a function of the adaptive Vineland-II scores (see also the reconstruction of average connectopy for neurotypical individuals only in Supplementary Fig. 11). This provides an estimation of the reduction in flatness observed within the S1 connectopy as functioning skills improve.

We have shown that as adaptive functioning increases, expressed by higher Vineland-II Daily Living scores, the color range within the gradient of the reconstructed connectopy becomes wider, reflecting a broader ROI to rest-of-brain differential connectivity pattern. Conversely, the gradient becomes flatter toward the other end of the Vineland scale, indicating lower functioning skills. The S1 connectopy of individuals with higher functioning skills, encompassing the broader range of differential connectivity, and the high model order that captures the more subtle changes of the connectopy, suggest higher functional differentiation in these individuals. Lower functional differentiation, on the other hand, is associated with lower adaptive ability, but this does not indicate the absence of a specific S1 function.

### Comparison between the connectopies of neurotypical and autistic individuals

Group comparisons of the S1 connectopies between neurotypical and autistic individuals did not yield any significant results for either hemisphere for both imaging measures (task and rest; see Supplementary Fig. 3). During the Hariri task, there were three TSM coefficients (y^2^, y^4^, and y^6^ [*p*-values: 0.017, 0.019, 0.025] for the left hemisphere) that were nominally significant but did not survive multiple comparisons correction (see Supplementary Material for visualization of the group comparison).

### Connectopic similarities and differences during rest and task

**Fig. 3:**
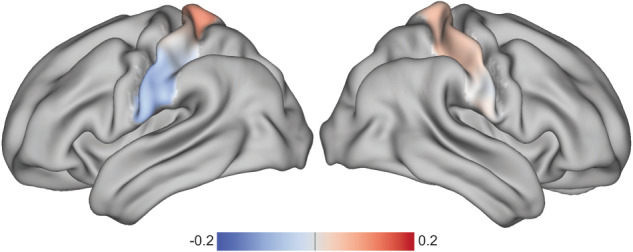
Differences between rfMRI and Hariri task S1 connectopies. Visualization of the connectopic differences after averaging the differences between the individual S1 connectopies during rfMRI and Hariri task (Connectopy_rfMRI_ – Connectopy_Hariri_).

To elucidate further the nature of the established association between connectopies and adaptive scores, we examined in more detail the differences between intrinsic functional connectivity (iFC) gradients during rest and task connectopies. Despite the dorsoventral axis of the connectopy being reproduced in both rest and task, we observed significant differences between the two. The TSM coefficients that were found to differ in the 192 participants for whom the scans of both conditions were available were 6 coefficients on the z-axis for the left hemisphere and 5 coefficients on the x- and y-axis for the right one (see Supplementary Material). The disparities between the rest and task connectopies are displayed in Fig. 3, highlighting a global, rather than a focal, pattern of differences along the dorsoventral axis of the S1.

**Fig. 4:**
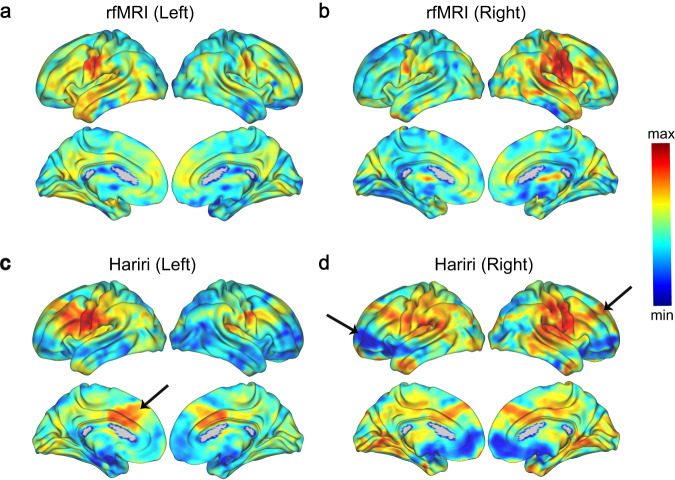
Projection maps. Projection maps of the S1 connectopies onto the entire brain during the rfMRI from the left and right hemisphere in (**a**) and (**b**) respectively, and during the Hariri task from the left and right hemisphere in (**c**) and (**d**) respectively. The arrows highlight the regions with connectivity profile of enhanced or diminished similarity with the S1 connectopy during task. These regions correspond respectively for the enhanced and diminished similarity cases to the anterior cingulate gyrus and frontal gyrus, and to a prefrontal component of the right hemisphere.

We additionally estimated the projections of the S1 connectopies onto the brain to highlight further the spatial components involved in the connectivity profile changes we observe. Projections during rest from the left and right hemisphere’s S1 connectopy are illustrated respectively in Fig. 4a, b and for the Hariri task in Fig. 4c, d. Overall, the color-gradients present in the left/right hemisphere reappear contralaterally in the opposite hemisphere, providing evidence that the two sensory strips are topographically connected. There is a general convergence between the two imaging measures (task and rest) (projections(left): Pearson’s *r* = 0.85 [*p*-value < .001], projections(right): Pearson’s *r* = 0.77 [*p*-value < .001]), apart from a frontal component – including frontal gyrus and anterior cingulate gyrus extending to the insular cortex (see Supplementary Figs. 12 and 13) – that seem to have enhanced similarity in its connectivity profile with S1 during task and a prefrontal component, on the opposing end, with a highlighted lack of similarity during task, more evident for the right hemisphere.

## Discussion

In this study, we investigated in a data-driven manner the fine-grained connectivity of the primary somatosensory cortex in the context of ASD and its potential cascading effects on key ASD traits. We detected an association between S1 connectopy during the emotional matching task and participants’ adaptive abilities in relation to their daily lives. We showed how S1 connectopy reflects variation in adaptive abilities, by demonstrating a gradient that follows the well-established somatotopic organization of a dorsomedial-to-ventrolateral axis (i.e., homunculus) in the left hemisphere of individuals and gradually flattens as their daily life adaptive abilities decline. We found no case-control differences between neurotypical and autistic individuals in either iFC or task gradients. Last, we examined the differences in S1 connectopies at rest and during task. The differences found there, present both in the S1 connectopies themselves and in their projection maps, led us to postulate that S1 is also influenced in a top-down manner, in such a way that its changes are then associated with enduring individual traits.

Our results provide evidence that the underlying biological mechanisms involved in the processing of lower-order sensory input are related to the higher-order behavioral atypicalities in ASD at the individual level. This extends recent discussions of the interplay between sensory atypicalities, such as under-responsiveness to touch and active seeking of sensational experiences, and social and communicative ones, as well as adaptive behavior [4, 6, 11]. In our work, we established a link with the individuals’ ability to adapt in their daily lives, as assessed by the Vineland-II Daily Living scale. An individual’s assessment on this scale is related to real-world outcomes, such as the likelihood of independent living and social competence, and thus acquires great significance for the impact on day-to-day matters of autistic individuals. The cascading effects of an aberrant connectivity profile within the S1 region presented here build on the idea suggested elsewhere [1] that potential disruptions in unimodal sensory information processing form the backbone of higher-order cortical abilities.

Notably, in this work we did not find a correlation between the S1 connectivity profile and sensory processing atypicalities, as reflected in the parent-reported SSP scores. There could be several reasons for this, from which the potential inadequacy of the TSM coefficients to effectively capture the gradual connectopy was excluded, as our GLM analysis on the raw connectopies replicated the same relationships as the ones revealed using the TSM coefficients. Our work is also susceptible to the potential limitations of SSP scores, such as the small number of items and the low dispersion across sensory modalities (tactile atypicalities corresponding to only a fraction of the items in the questionnaire), which may not be able to capture the large heterogeneity of sensory phenotypes in ASD [37]. In addition, the large age range considered in this work may further complicate matters when considering the developmental component of ASD sensory phenotypes, further limiting the power of the current sample and thus our ability to capture a potentially relevant relationship. Of note, previous studies on the neurobiology of sensory profiles report have used only a subset of SSP items and Sensory over-responsivity scores [38], as it has been argued that SSP, although useful for clinical image formulation, may correlate less with brain structure than direct assessment [39].

Our estimated S1 connectopies followed dorsomedial-to-ventrolateral axis, as expected, consistent with the established somatotopic organization of the region [16, 40, 41]. However, by delineating in more detail the profile of connectivity changes within the S1 region at specific points on the Vineland-II Daily Living scale we were able to show that the lower the adaptive skills of the individual, the less functionally differentiated the gradient describing them. On the contrary, the better an individual functions in daily life, the broader the color range of their S1 gradients seems to become, which translates into higher variability of connectivity profiles within this region and potentially stronger differentiation in the associated pattern of connectivity. Our findings are consistent with a growing body of evidence suggesting that ASD occurs due to altered communication between brain regions, and, in particular, from reduced functional segregation of large-scale brain networks [42, 43]. A recent study in which the authors used a global connectopic mapping approach has also shown that the segregation between sensory systems and unimodal, as well as transmodal, convergence regions appears to be diminished in ASD [42]. In our work, we link the notion of a varying degree of functional differentiation within S1 to a varying degree of adaptive behavior in the daily life of individuals, predominantly affected in ASD.

In addition to the discussion of how and whether the focus on the average patient may actually mask inter-individual differences in psychiatry [44], the lack of case-control differences in our results points to the known heterogeneity of ASD [20] and underscores the need to examine individual variation instead. Although the disrupted cortical connectivity theory is one of the prominent theories related to ASD and its etiology, results about the exact localization and the identification of the mechanisms involved (what type of connectivity disruption, e.g., hyper- or hypo-connectivity, and its behavioral link) involved have been mixed and inconclusive [45, 46]. Besides the heterogeneity characterizing ASD and potentially being the main reason for the above-mentioned elusiveness of findings, there is also the assumption of piecewise constant connectivity that most studies have relied on so far, neglecting the functional heterogeneity in terms of connectivity within specific brain regions [19]. Nonetheless, our work highlights the need for a data-driven description that takes place at the individual level, characterizes connectivity changes within a region in a gradual manner and examines their association with core ASD phenotypes.

We also found topographic differences in S1 between rest and the emotional matching paradigm, Hariri. This raises the speculation of a disrupted cortical hierarchy due to higher-order processes occurring during emotion recognition that would require the presence of non-unilateral connections in S1 which stands in alignment with a fundamental but often neglected feature of the somatosensory cortex; the presence of massive descending projections that even outnumber ascending somatic sensory pathways [47]. Additionally, the existence of a frontal component in the projection maps of the S1 connectopies present during task, and not during rest, supports the idea that S1 is not solely responsible for the low-level processing of sensory stimuli. The frontal component is mostly located around the area of cingulate gyrus that has been found to have great influence on social behavior [48], as well as on regulation of emotional processing [49], and is involved in both the sensory and social atypicalities observed in ASD [50]. S1 projections to insula, brain region which is well known for its contribution to the social emotions experienced when interacting with others, further strengthens the speculation of the S1 involvement in processing and/or generation of fearful emotions [10]. While differences in the brain regions recruited by autistic individuals for the purposes of multisensory integration in the context of emotion recognition have been found before [50], our results highlight S1 as being implicated in the activities of daily life and could be beneficial, not only for ASD, but also for conditions characterized by co-occurring emotional and sensory difficulties.

Taken together, our findings suggest a potential overarching link between higher- and lower-level processes in the brain, relying on long-range bottom-up, as well as top-down, connections, whose aberrant communication will be reflected on someone’s daily life functioning skills. Our findings are consistent with studies suggesting that atypical sensory features in ASD are a consequence of both bottom-up, and top-down processing differences [38] and highlight a potential transdiagnostic value of S1 in cases where sensory distress co-occurs with higher-order cognitive atypicalities.

Although we provide convincing evidence for the validity of the S1 connectopies using two individual datasets and imaging measures, there is a certain limitation that needs to be highlighted. We use the Harvard–Oxford anatomical atlas, which, although well established in our field, remains a probabilistic atlas that could potentially lead to signal contamination from neighboring brain regions and interfere with the alignment between the actual functional boundaries of the selected region for S1 and the boundaries used in this work.

To understand further what we have studied here, we could benefit from applying connectopic mapping to different sensory modalities, such as auditory or face processing of the somatosensory – in the latter case higher spatial resolution would be required. In addition, an important aspect of sensory atypicalities that we cannot address by looking at a single time point alone is their developmental effect. This could be further investigated by using data from subsequent time points from the LEAP dataset. Assessing inter-individual differences in a paired design may prove more sensitive concerning the effects examined here compared to a cross-sectional analysis. Lastly, because sensory hyper-/hypo-responsivity is not unique to ASD (but is albeit more common in this population than in other developmental disorders), the relationship examined in the current work could be explored in other clinical populations.

In this study, we showed that individual variation present in the dorsoventral connectopies within the primary somatosensory cortex is associated with dimensional measures accessing individuals’ adaptive skills variation in daily life in autistic individuals, as well as neurotypicals. Furthermore, differences between S1 connectopies and their projection maps at rest and during task suggest a modulatory role of primary somatosensory cortex within a possible top-down regulation of the low-level somatosensory processing framework. The findings of this study lay the foundation for developing more specific hypotheses regarding the mechanisms of sensory processing dysfunction in ASD and provide an opportunity for earlier identification of phenotypic features that may aid in the diagnosis of ASD, but also in other conditions characterized by co-occurring sensory and emotional stress.

## Supplementary information

Supplemental Material
