# Supplementary material for: Fine-grained topographic organization within somatosensory cortex during resting-state and emotional face-matching task and its association with ASD traits
Source: Transl Psychiatry. 2023 Jul 27;13:270. doi: 10.1038/s41398-023-02559-3 (PMC10374902; doi:10.1038/s41398-023-02559-3)
Supplement: Supplementary file 1 — Supplemental Material [file 41398_2023_2559_MOESM1_ESM.docx]

**Supplementary Material**

Contents

[Methods 1](#_Toc117538591)

[Fmri data acquisition 1](#_Toc117538592)

[Data preparation for connectopic mapping 2](#_Toc117538593)

[Results 3](#_Toc117538594)

[Model Selection 3](#_Toc117538595)

[Visualization of the somatosensory connectopies for neurotypicals and autistic individuals 4](#_Toc117538596)

[Association between assessment scores and spatial coefficients of connectopies 5](#_Toc117538597)

[GLM analysis on raw connectopies during the Hariri task 8](#_Toc117538598)

[Reconstruction of average connectopy for neurotypical individuals during the Hariri task 9](#_Toc117538599)

[Differences between rfMRI and Hariri task connectopies 9](#_Toc117538600)

[S1 projections 10](#_Toc117538601)

[TSM modeling on eroded connectopies 10](#_Toc117538602)

[Implementation 11](#_Toc117538603)

[References 11](#_Toc117538604)

# Methods

## Fmri data acquisition

MRI data were acquired on different 3T scanners at multiple sites in Europe: General Electric MR750 (GE Medical Systems, Milwaukee, WI, USA) at Institute of Psychiatry, Psychology and Neuroscience, King’s College London, United Kingdom (KCL); Siemens Magnetom Skyra (Siemens, Erlangen, Germany) at Radboud University Nijmegen Medical Centre, the Netherlands (RUNMC); Siemens Magnetom Verio (Siemens, Erlangen, Germany) at the Autism Research Centre at the University of Cambridge, United Kingdom (UCAM); Philips 3T Achieva (Philips Healthcare Systems, Best, The Netherlands) at University Medical Centre Utrecht, the Netherlands (UMCU); and Siemens Magnetom Trio (Siemens, Erlangen, Germany) at Central Institute of Mental Health, Mannheim, Germany (CIMH) (see Supplementary Material). Structural images were obtained using a 5.5-minute MPRAGE sequence (TR=2300ms, TE=2.93ms, T1=900ms, voxels size=1.1x1.1x1.2mm, flip angle=9°, matrix size=256x256, FOV=270mm, 176 slices). An eight-to-ten minute resting-state fMRI (rfMRI) scan was acquired using a multi-echo planar imaging (ME-EPI) sequence; TR=2300ms, TE~12ms, 31ms, and 48ms (slight variations are present across centers), flip angle=80°, matrix size=64x64, in-plane resolution=3.8mm, FOV=240mm, 33 axial slices, slice thickness/gap=3.8mm/0.4mm, volumes=200 (UMCU), 215 (KCL,CIMH), or 265 (RUNMC, UCAM). Participants were instructed to relax and fixate on a cross presented on the screen for the duration of the rfMRI scan (for further details see [1]).

## Data preparation for connectopic mapping

For rs-fMRI, we excluded the participants for which there were more than 5000 voxels with invalid values (inf/nan) within the region of our interest (n=8). We then shrank the ROI by removing the voxels in which there was at least one participant that had invalid values and used this as the common roi for estimating all individual connectopies. We further excluded the participants whose connectopies had spatial correlation with the HCP reference connectopy less than 0.5 (n=99).

For the Hariri task, we used the ROI we obtained during rs-fMRI and we excluded the participants that had any missing value within it (n=38). One extra participant was also excluded due to missing clinical information.


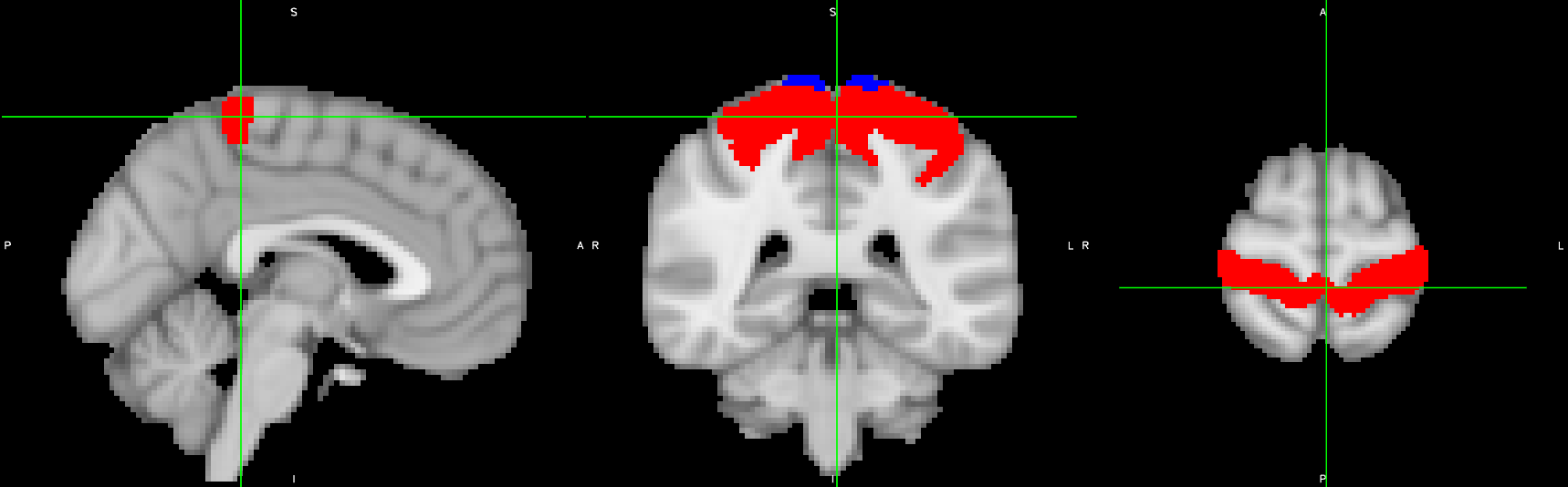


Figure 1 Obtained ROI after the procedure described above is displayed in red on top of the original ROI. The blue part seen here is the part for which there were invalid values for at least one participant, and hence removed.

# Results

## Model Selection


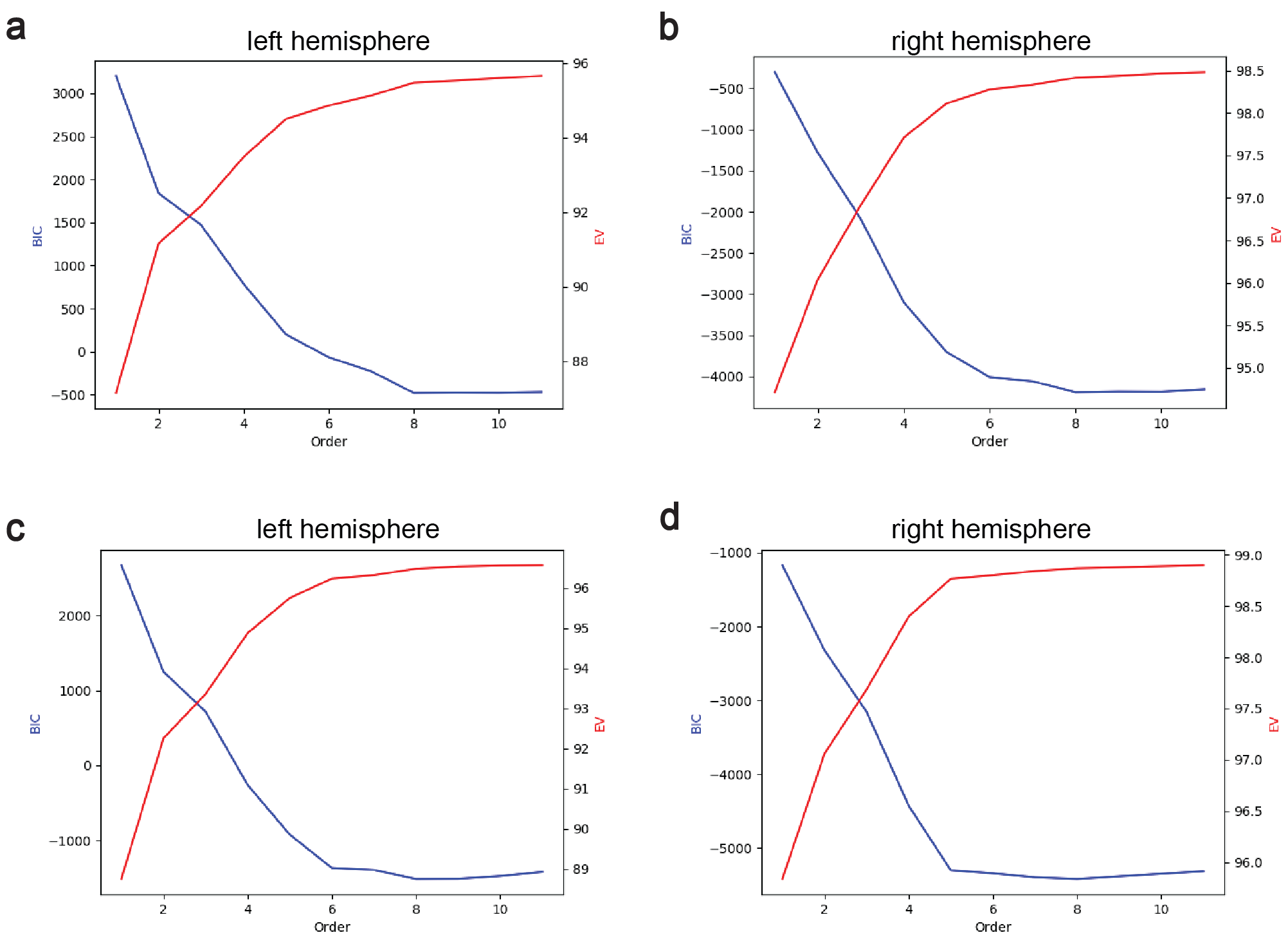


Figure 2 **Model order selection process.** The model order that simultaneously minimizes BIC and maximizes EV was selected. A. and B. show the BIC and EV as a function of model order during rs-fMRI for the left and right hemispheres, respectively, while C. and D. show the same quantities for the task condition and for the left and right hemispheres, respectively. Above the 6^th^ model order, the model was not drastically improved for all four conditions. Therefore, in order not to increase the complexity of our model without achieving a large benefit that would justify its use, we decided to use this model order.

## Visualization of the somatosensory connectopies for neurotypicals and autistic individuals


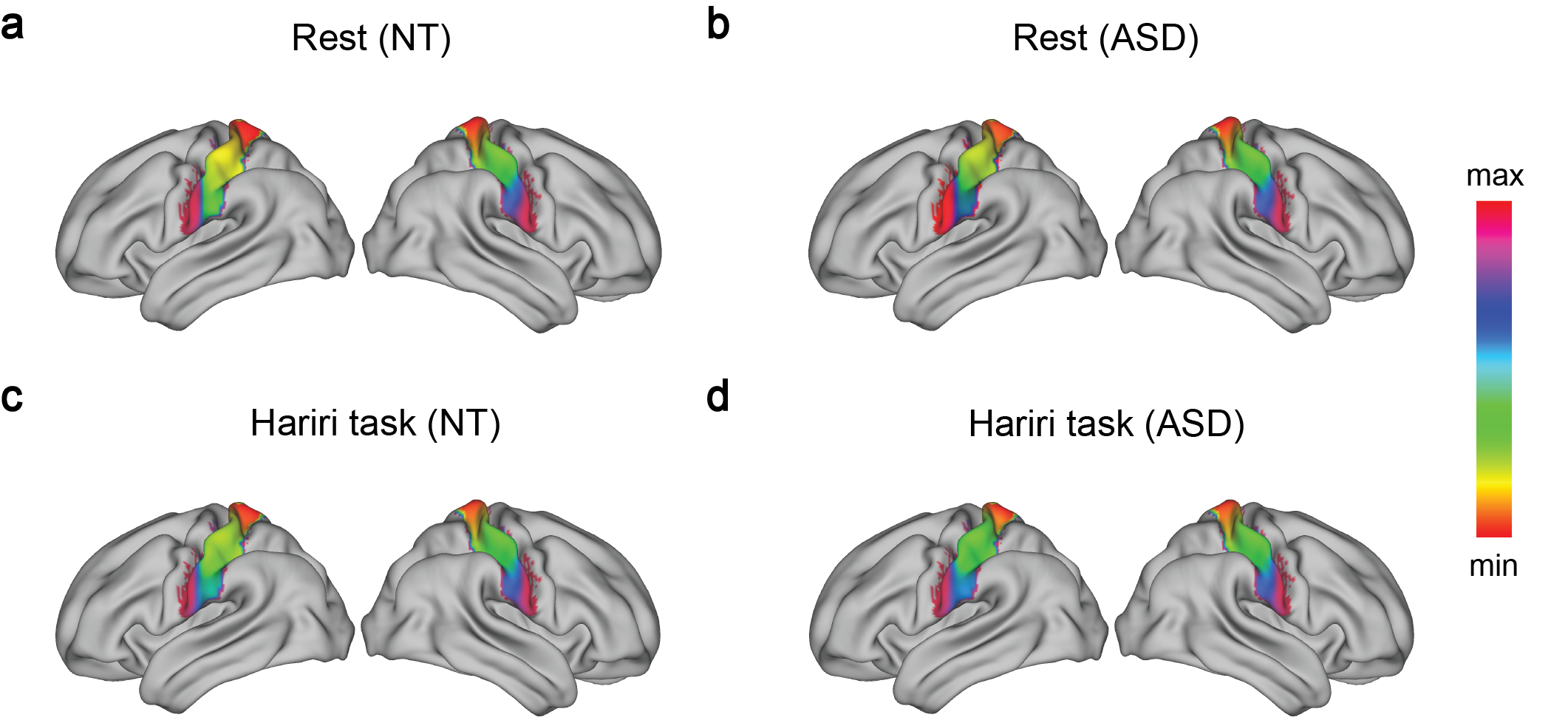


Figure 3 **Visualization of the average S1 connectopies for rest and task conditions separately for neurotypicals (NT) and autistic individuals (ASD).**

## Association between assessment scores and spatial coefficients of connectopies

| **Scores** | **x** | **y** | **z** | **x2** | **y2** | **z2** | **x3** | **y3** | **z3** | **x4** | **y4** | **z4** | **x5** | **y5** | **z5** | **x6** | **y6** | **z6** |
| --- | --- | --- | --- | --- | --- | --- | --- | --- | --- | --- | --- | --- | --- | --- | --- | --- | --- | --- |
| **SRS-2** | -0.03 | 0.07 | 0.02 | 0.02 | 0.11 | -0.08 | -0.03 | -0.04 | 0.06 | -0.02 | -0.09 | 0.05 | 0.06 | 0.04 | -0.04 | -0.03 | 0.08 | -0.05 |
| **SSP** | 0.02 | -0.16 | 0.03 | -0.11 | -0.08 | 0.21 | 0.06 | 0.16 | -0.07 | 0.02 | 0.06 | -0.17 | -0.1 | -0.16 | 0.03 | 0.07 | -0.05 | 0.11 |
| **RBS-R** | 0 | 0.14 | 0.03 | 0.01 | -0.03 | -0.14 | -0.03 | -0.12 | -0.03 | 0.05 | 0.05 | 0.15 | 0.06 | 0.12 | 0.07 | -0.09 | -0.05 | -0.07 |
| **ADI-R Social** | 0.14 | 0.19 | -0.1 | 0.26 | 0.03 | -0.19 | -0.07 | -0.18 | 0.1 | -0.2 | -0.04 | 0.21 | 0.01 | 0.17 | -0.1 | 0.12 | 0.04 | -0.21 |
| **ADI-R Comm.** | 0.15 | 0.27 | -0.09 | 0.11 | 0.06 | -0.14 | -0.03 | -0.11 | 0.14 | -0.1 | -0.02 | 0.17 | -0.02 | 0.1 | -0.15 | 0.08 | 0.01 | -0.21 |
| **ADI-R RBS** | 0.12 | 0.23 | -0.04 | 0.12 | 0.06 | -0.14 | -0.05 | -0.17 | 0.06 | -0.11 | -0.05 | 0.18 | 0 | 0.2 | -0.05 | 0.08 | 0.04 | -0.17 |
| **Vineland-II Comm.** | 0 | -0.12 | 0.01 | -0.09 | -0.1 | 0.14 | 0.02 | 0.14 | -0.12 | 0.1 | 0.08 | -0.11 | -0.01 | -0.1 | 0.12 | -0.07 | -0.08 | 0.12 |
| **Vineland-II D.Liv.** | -0.02 | -0.18 | 0.11 | -0.18 | -0.05 | **0.28*** | -0.03 | 0.17 | -0.16 | 0.14 | 0.04 | **-0.26*** | 0.06 | -0.14 | 0.11 | -0.12 | -0.04 | 0.21 |
| **Vineland-II Soc.** | 0.07 | -0.12 | 0.13 | -0.12 | -0.06 | 0.24 | -0.12 | 0.14 | -0.22 | 0.11 | 0.05 | -0.23 | 0.1 | -0.13 | 0.18 | -0.1 | -0.04 | 0.24 |
| **Vineland-II ABC** | 0.02 | -0.15 | 0.08 | -0.18 | -0.08 | 0.23 | -0.02 | 0.18 | -0.19 | 0.14 | 0.09 | -0.2 | 0.02 | -0.15 | 0.18 | -0.09 | -0.09 | 0.23 |

Figure 4 **Correlation analysis between the TSM parameters of the S1 primary connectopies during the Hariri task and the ASD clinical/behavioral scores for the left hemisphere.** Heatmap correlations [*: used to denote statistical significance after controlling and correcting multiple comparisons correction, SRS-2: Social Responsiveness Scale-2, RBS-R: Repetitive Behaviors Scale-Revised, ADI-R: Autism Diagnostic Interview-Revised, Vineland-II Comm.: Vineland-II Communication, Vineland-II D.Liv.: Vineland-II Daily Living, Vineland-II Soc.: Vineland-II Socialisation, Vineland-II-ABC: Vineland-II Adaptive Behaviour Composite (ABC) standard score.

| **Scores** | **x** | **y** | **z** | **x2** | **y2** | **z2** | **x3** | **y3** | **z3** | **x4** | **y4** | **z4** | **x5** | **y5** | **z5** | **x6** | **y6** | **z6** |
| --- | --- | --- | --- | --- | --- | --- | --- | --- | --- | --- | --- | --- | --- | --- | --- | --- | --- | --- |
| **SRS-2** | 0.03 | 0.09 | 0.05 | 0.03 | 0.04 | 0.01 | -0.09 | 0.07 | 0.01 | -0.05 | -0.06 | -0.04 | 0.08 | -0.11 | -0.02 | 0.06 | 0.07 | 0.02 |
| **SSP** | -0.01 | -0.18 | -0.05 | -0.02 | 0.01 | -0.06 | 0.05 | 0.03 | -0.11 | 0.05 | 0.01 | 0.03 | -0.06 | 0.01 | 0.11 | -0.08 | -0.01 | 0.03 |
| **RBS-R** | -0.03 | 0.03 | 0.03 | -0.07 | -0.01 | 0.12 | 0.02 | 0.08 | 0 | 0.05 | 0 | -0.1 | -0.02 | -0.13 | 0 | -0.04 | 0 | 0.07 |
| **ADI-R Social** | -0.05 | 0.03 | 0.09 | -0.09 | 0.15 | 0.03 | 0.12 | 0.08 | -0.02 | 0.08 | -0.13 | -0.06 | -0.14 | -0.11 | 0.02 | -0.09 | 0.12 | 0.06 |
| **ADI-R Comm.** | 0.06 | 0.09 | 0.05 | -0.16 | 0.08 | 0.11 | -0.02 | 0.01 | 0.03 | 0.12 | -0.05 | -0.07 | -0.03 | -0.03 | 0 | -0.11 | 0.03 | 0.06 |
| **ADI-R RBS** | -0.06 | 0.1 | 0.06 | 0.05 | 0.01 | 0.11 | 0.06 | -0.04 | 0.07 | -0.05 | -0.03 | -0.09 | -0.04 | -0.01 | -0.11 | 0.04 | 0.02 | -0.02 |
| **Vineland-II Comm.** | -0.05 | 0.18 | -0.09 | -0.17 | -0.19 | 0.09 | -0.01 | -0.2 | -0.01 | 0.16 | 0.24 | 0.04 | -0.01 | 0.25 | 0.09 | -0.12 | -0.25 | 0.03 |
| **Vineland-II D.Liv.** | -0.02 | 0.09 | -0.01 | 0.02 | -0.16 | 0.12 | -0.04 | -0.19 | -0.06 | -0.01 | 0.15 | -0.06 | 0.05 | 0.26 | 0.08 | 0.01 | -0.15 | 0.07 |
| **Vineland-II Soc.** | -0.01 | 0.06 | 0.02 | -0.06 | -0.04 | 0 | -0.02 | -0.1 | -0.06 | 0.05 | 0.07 | 0 | 0.02 | 0.14 | 0.06 | -0.04 | -0.08 | 0.03 |
| **Vineland-II ABC** | -0.04 | 0.1 | -0.04 | -0.07 | -0.14 | 0.09 | -0.02 | -0.14 | -0.04 | 0.07 | 0.17 | -0.03 | 0.02 | 0.21 | 0.04 | -0.05 | -0.17 | 0.02 |

Figure 5 **Correlation analysis between the TSM parameters of the S1 primary connectopies during the Hariri task and the ASD clinical/behavioral scores for the right hemisphere.** Heatmap correlations [*: used to denote statistical significance after controlling and correcting multiple comparisons correction, SRS-2: Social Responsiveness Scale-2, RBS-R: Repetitive Behaviors Scale-Revised, ADI-R: Autism Diagnostic Interview-Revised, Vineland-II Comm.: Vineland-II Communication, Vineland-II D.Liv.: Vineland-II Daily Living, Vineland-II Soc.: Vineland-II Socialisation, Vineland-II-ABC: Vineland-II Adaptive Behaviour Composite (ABC) standard score.

| **Scores** | **x** | **y** | **z** | **x2** | **y2** | **z2** | **x3** | **y3** | **z3** | **x4** | **y4** | **z4** | **x5** | **y5** | **z5** | **x6** | **y6** | **z6** |
| --- | --- | --- | --- | --- | --- | --- | --- | --- | --- | --- | --- | --- | --- | --- | --- | --- | --- | --- |
| **SRS-2** | 0.03 | 0.08 | 0.05 | 0.03 | 0.09 | -0.03 | -0.03 | -0.05 | 0.01 | -0.03 | -0.1 | 0.01 | 0.02 | 0.04 | -0.04 | 0.02 | 0.1 | -0.02 |
| **SSP** | -0.01 | -0.08 | -0.08 | 0 | -0.06 | 0.06 | -0.01 | 0.07 | 0.04 | 0.01 | 0.07 | -0.04 | 0.03 | -0.05 | -0.04 | -0.03 | -0.07 | 0 |
| **RBS-R** | 0.07 | 0.15 | 0.04 | -0.05 | 0.11 | -0.06 | -0.03 | -0.07 | -0.01 | 0.05 | -0.07 | 0.02 | 0 | 0.04 | 0 | -0.01 | 0.05 | -0.01 |
| **ADI-R Social** | 0.06 | 0 | -0.01 | 0.1 | -0.09 | 0 | -0.06 | -0.07 | -0.09 | -0.03 | 0.04 | -0.01 | 0.04 | 0.08 | 0.11 | -0.01 | -0.02 | 0.06 |
| **ADI-R Comm.** | 0.05 | 0.06 | -0.08 | 0.14 | 0.01 | -0.07 | -0.06 | -0.12 | 0.04 | -0.06 | -0.04 | 0.05 | 0.04 | 0.09 | -0.01 | 0.01 | 0.04 | -0.02 |
| **ADI-R RBS** | 0 | 0.11 | 0.04 | 0.03 | 0.14 | -0.01 | -0.11 | -0.2 | -0.02 | 0 | -0.17 | -0.03 | 0.1 | 0.18 | 0.02 | -0.05 | 0.16 | 0.04 |
| **Vineland-II Comm.** | 0.09 | 0.12 | -0.09 | -0.17 | -0.08 | -0.02 | 0.01 | 0.03 | 0 | 0.15 | 0.16 | 0.06 | -0.03 | -0.05 | 0.05 | -0.07 | -0.17 | -0.01 |
| **Vineland-II D.Liv.** | 0.09 | 0.1 | -0.13 | -0.2 | -0.12 | -0.08 | 0.06 | 0.08 | 0.05 | 0.16 | 0.23 | 0.12 | -0.08 | -0.08 | 0.01 | -0.04 | -0.25 | -0.06 |
| **Vineland-II Soc.** | 0.01 | 0.07 | -0.11 | -0.13 | -0.08 | -0.06 | 0.08 | 0.01 | 0.06 | 0.09 | 0.16 | 0.1 | -0.09 | -0.02 | -0.02 | -0.01 | -0.16 | -0.06 |
| **Vineland-II ABC** | 0.07 | 0.11 | -0.13 | -0.17 | -0.1 | -0.06 | 0.06 | 0.03 | 0.05 | 0.14 | 0.2 | 0.11 | -0.08 | -0.04 | 0.01 | -0.03 | -0.21 | -0.06 |

| **Scores** | **x** | **y** | **z** | **x2** | **y2** | **z2** | **x3** | **y3** | **z3** | **x4** | **y4** | **z4** | **x5** | **y5** | **z5** | **x6** | **y6** | **z6** |
| --- | --- | --- | --- | --- | --- | --- | --- | --- | --- | --- | --- | --- | --- | --- | --- | --- | --- | --- |
| **SRS-2** | -0.01 | 0.04 | 0.09 | 0 | 0.07 | 0.04 | 0 | 0.05 | -0.05 | -0.02 | -0.08 | -0.07 | 0.01 | -0.09 | 0.04 | 0.02 | 0.07 | 0.07 |
| **SSP** | 0.02 | -0.03 | -0.12 | 0 | -0.04 | -0.04 | 0 | -0.05 | 0.02 | 0.04 | 0.05 | 0.03 | -0.02 | 0.07 | 0 | -0.04 | -0.04 | 0 |
| **RBS-R** | -0.04 | 0.02 | 0.06 | 0.02 | 0.04 | 0.08 | 0.06 | -0.02 | 0.01 | -0.05 | -0.03 | -0.05 | -0.03 | 0.03 | 0 | 0.05 | 0.03 | 0.03 |
| **ADI-R Social** | -0.06 | 0.08 | -0.03 | 0.04 | -0.03 | -0.01 | 0.04 | -0.11 | -0.04 | 0.01 | 0.01 | 0.01 | -0.03 | 0.13 | 0.07 | -0.02 | -0.02 | 0.04 |
| **ADI-R Comm.** | -0.06 | 0.11 | -0.08 | 0.1 | 0 | -0.07 | 0.03 | -0.11 | 0.03 | -0.03 | -0.01 | 0.07 | -0.02 | 0.09 | -0.01 | 0.01 | 0.01 | -0.04 |
| **ADI-R RBS** | 0.01 | 0.1 | 0.03 | 0.04 | 0.13 | 0.01 | 0.03 | -0.14 | 0.02 | -0.04 | -0.13 | -0.04 | -0.03 | 0.11 | -0.03 | 0.02 | 0.12 | 0 |
| **Vineland-II Comm.** | -0.05 | -0.04 | -0.03 | -0.16 | -0.1 | 0.05 | 0.02 | 0.07 | -0.05 | 0.1 | 0.15 | 0.01 | -0.01 | -0.02 | 0.07 | -0.07 | -0.15 | 0.04 |
| **Vineland-II D.Liv.** | -0.04 | -0.05 | 0 | -0.2 | -0.09 | 0.04 | -0.02 | 0.15 | -0.06 | 0.12 | 0.16 | 0 | 0.03 | -0.11 | 0.07 | -0.07 | -0.16 | 0.04 |
| **Vineland-II Soc.** | -0.02 | -0.03 | -0.03 | -0.18 | -0.05 | -0.02 | -0.02 | 0.07 | -0.03 | 0.12 | 0.11 | 0.06 | 0.02 | -0.03 | 0.05 | -0.08 | -0.12 | 0.01 |
| **Vineland-II ABC** | -0.04 | -0.05 | -0.03 | -0.19 | -0.09 | 0.03 | -0.01 | 0.1 | -0.04 | 0.13 | 0.16 | 0.03 | 0.01 | -0.05 | 0.07 | -0.08 | -0.16 | 0.03 |

Figure 6 **Correlation analysis between the TSM parameters of the S1 primary connectopies during rs-fMRI and the ASD clinical/behavioral scores for the left hemisphere.** Heatmap correlations [*: used to denote statistical significance after controlling and correcting multiple comparisons correction, SRS-2: Social Responsiveness Scale-2, RBS-R: Repetitive Behaviors Scale-Revised, ADI-R: Autism Diagnostic Interview-Revised, Vineland-II Comm.: Vineland-II Communication, Vineland-II D.Liv.: Vineland-II Daily Living, Vineland-II Soc.: Vineland-II Socialisation, Vineland-II-ABC: Vineland-II Adaptive Behaviour Composite (ABC) standard score.

Figure 7 **Correlation analysis between the TSM parameters of the S1 primary connectopies during rs-fMRI and the ASD clinical/behavioral scores for the right hemisphere.** Heatmap correlations [*: used to denote statistical significance after controlling and correcting multiple comparisons correction, SRS-2: Social Responsiveness Scale-2, RBS-R: Repetitive Behaviors Scale-Revised, ADI-R: Autism Diagnostic Interview-Revised, Vineland-II Comm.: Vineland-II Communication, Vineland-II D.Liv.: Vineland-II Daily Living, Vineland-II Soc.: Vineland-II Socialisation, Vineland-II-ABC: Vineland-II Adaptive Behaviour Composite (ABC) standard score.

## GLM analysis on raw connectopies during the Hariri task


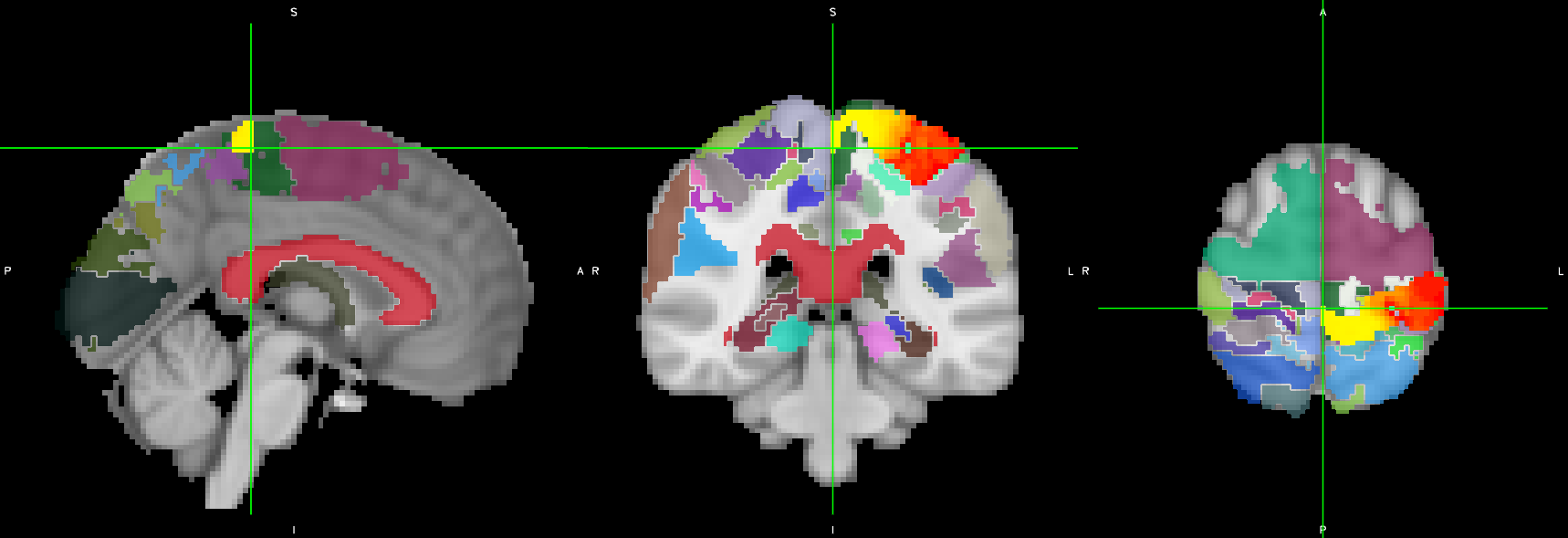


Figure 8 T-maps for Vineland-II Daily living scores, superimposed on the Juelich atlas

(Only the significant voxels after correction are shown in the red-yellow color range).


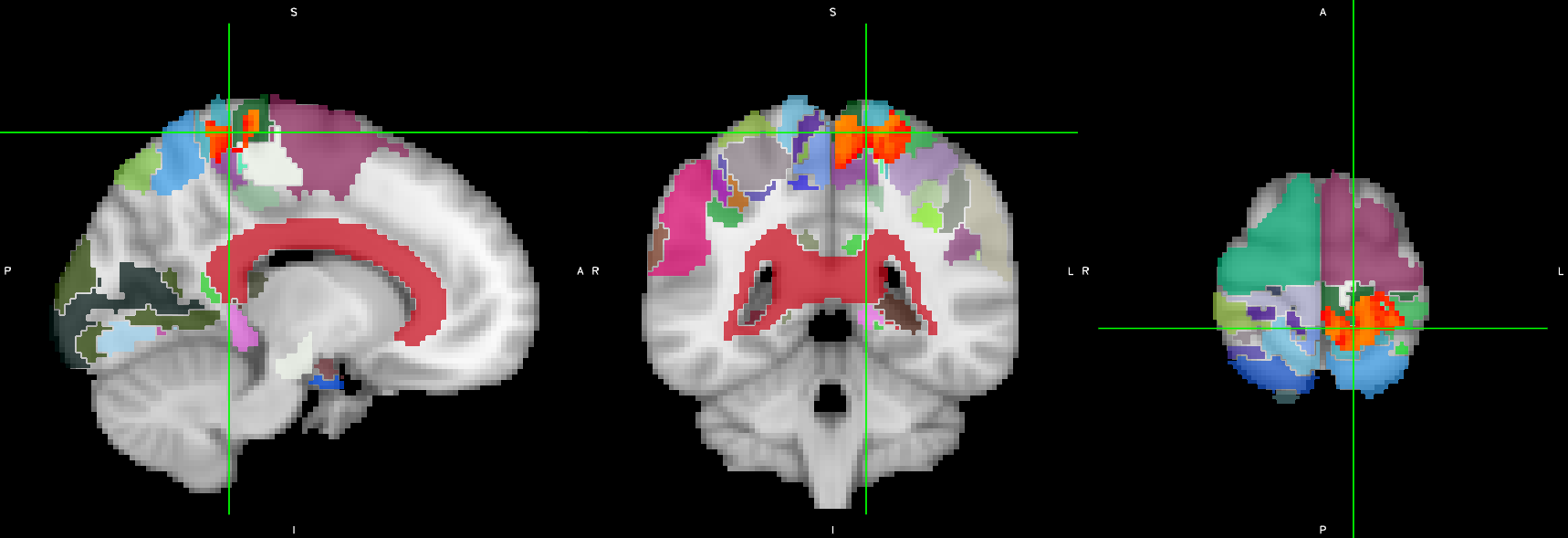


Figure 9 T-maps for Vineland-II Socialization scores, superimposed on the Juelich atlas

(Only the significant voxels after correction are shown in the red-yellow color range).


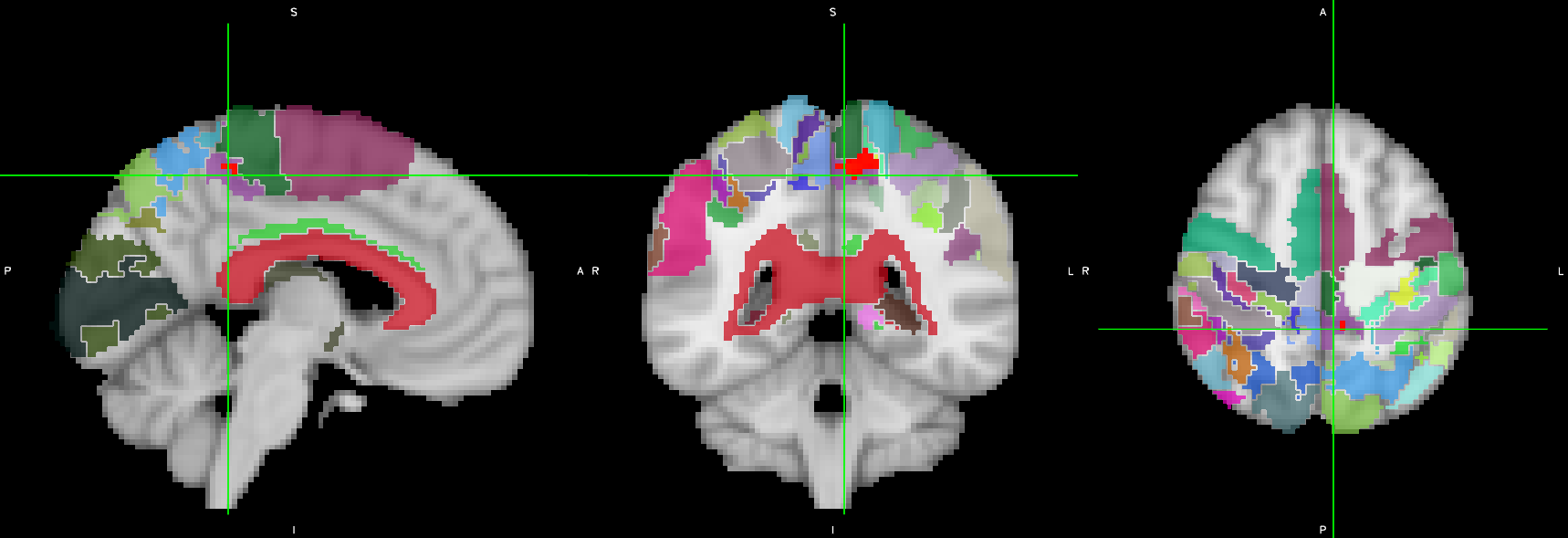


Figure 10 T-maps for Vineland-II Communication scores, superimposed on the Juelich atlas

(Only the significant voxels after correction are shown in the red-yellow color range).

## Reconstruction of average connectopy for neurotypical individuals during the Hariri task


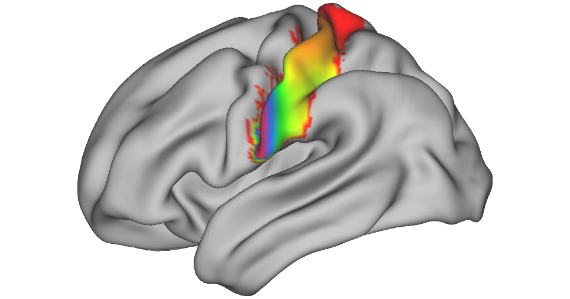


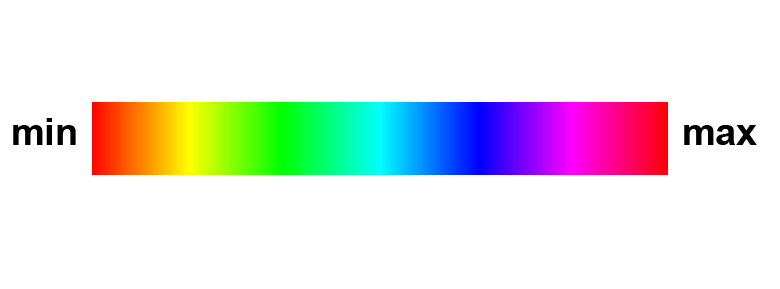


Figure 11 Reconstruction of average connectopy for neurotypical individuals for which the Vineland-II Daily Living scores are available during the Hariri task (left hemisphere).

## Differences between rfMRI and Hariri task connectopies

TSM parameters that were found to differ between rest and task connectopies for left and right hemisphere, along with their respective corrected p-values.

| Hemisphere | Left | Right |
| --- | --- | --- |
| TSM parameters | z, z^2^, z^3^, z^4^, z^5^, z^6^ | x^2^, x^4^, y^4^, x^6^, y^6^ |
| p-values | 0.0011, 0.0053, 0.0003, 0.0002, 0.0075, 0.0002 | 3e-06, 0.0001, 0.0073, 0.0028, 0.0038 |

## S1 projections


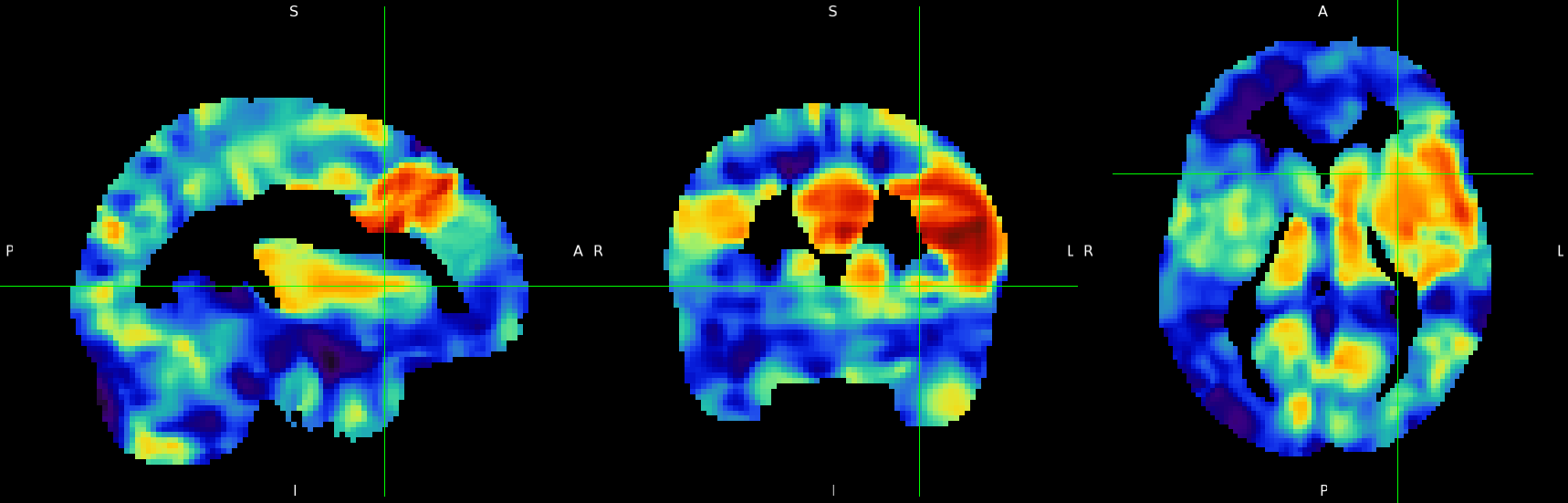


Figure 12 Slice view visualization of the projection maps from the left hemisphere during the Hariri task. Extension of the Figure 4 from the main text highlighting the enhanced similarity of S1’s connectivity profile with that of the insular cortex. Red colors indicates higher similarity, whereas blue ones reduced.


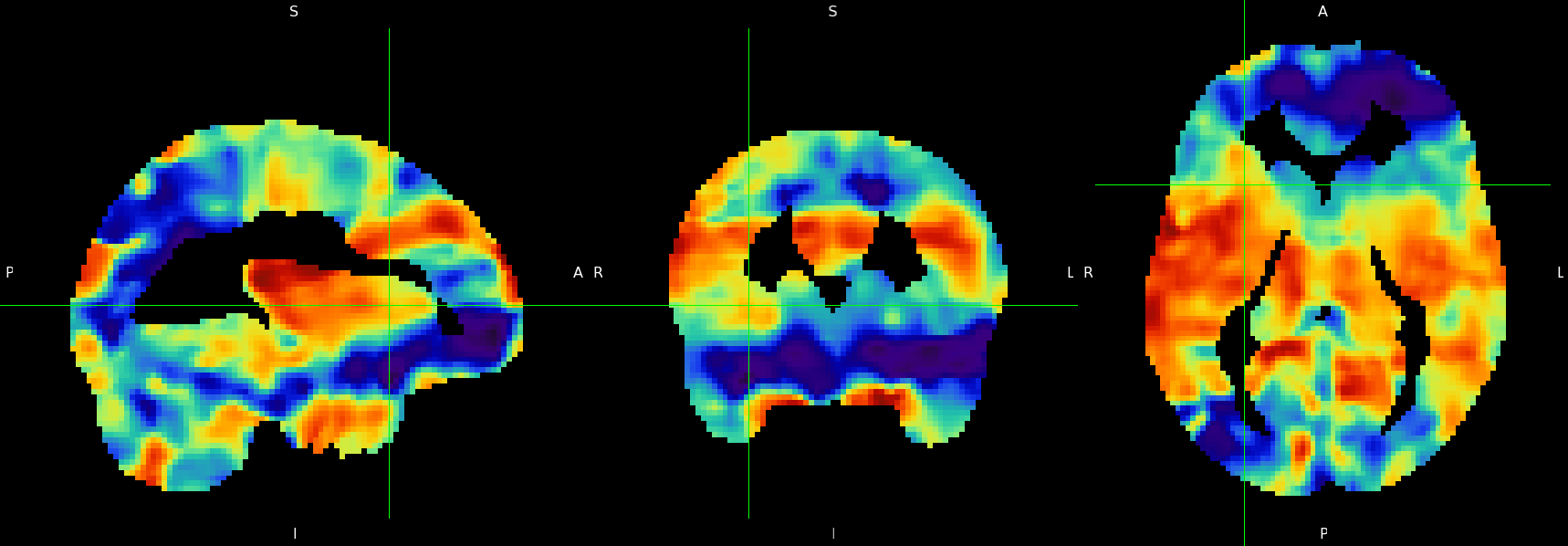


Figure 13 Slice view visualization of the projection maps from the right hemisphere during the Hariri task. Extension of the Figure 4 from the main text highlighting the enhanced similarity of S1’s connectivity profile with that of the insular cortex. Red colors indicates higher similarity, whereas blue ones reduced.

## TSM modeling on eroded connectopies

Figure 14 Distribution density of TSM coefficient values for coefficient z^2^ on the left and z^4^ on the right of the original connectopies from 50 randomly selected individuals contrasted with their eroded connectopies. Eroded connectopies were generated using fsl erode (fslmaths -ero) and a 3D kernel 5x5x5.


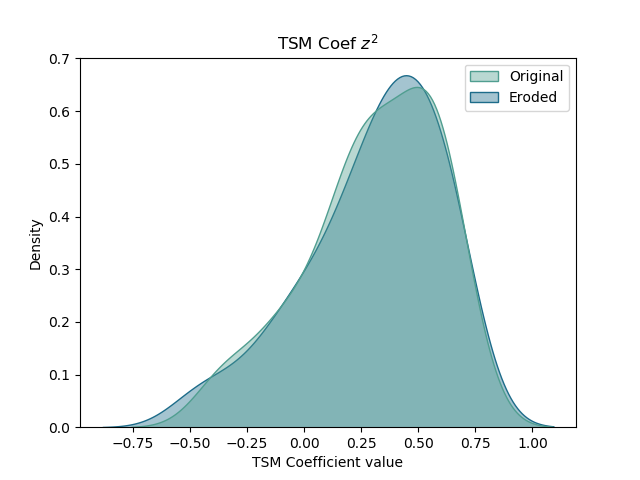

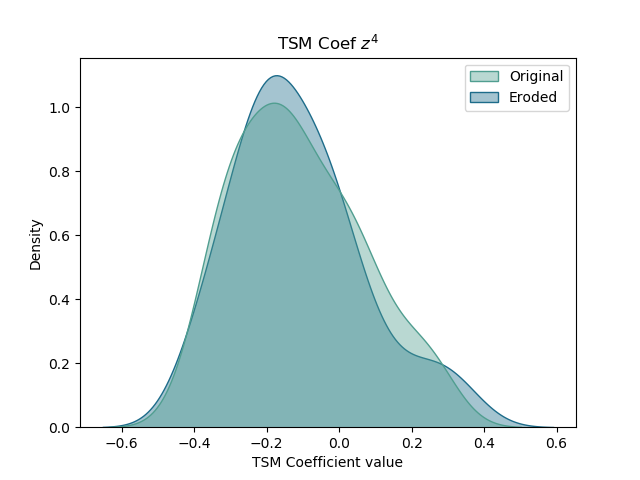


# Implementation

For the implementation of all the statistical tests, the Python module statsmodels (v0.9.0) and scipy.stats (v1.2.1) was used (group comparison between neurotypical and Autistic individuals s performed using ttest_ind, and group comparison between rest and Hariri using ttest_rel).

# References

[1] Oldehinkel M, Mennes M, Marquand A, Charman T, Tillmann J, Ecker C, *et al.* (2019): Altered Connectivity Between Cerebellum, Visual, and Sensory-Motor Networks in Autism Spectrum Disorder: Results from the EU-AIMS Longitudinal European Autism Project. *Biol Psychiatry Cogn Neurosci Neuroimaging* 4: 260–270.

[2] Haak K v., Marquand AF, Beckmann CF. Connectopic mapping with resting-state fMRI. Neuroimage. 2018; 170: 83–94.
